# Fluid-Structure Interaction Simulation of an Intra-Atrial Fontan Connection

**DOI:** 10.3390/biology9120412

**Published:** 2020-11-24

**Authors:** Elaine Tang, Zhenglun (Alan) Wei, Mark A. Fogel, Alessandro Veneziani, Ajit P. Yoganathan

**Affiliations:** 1 School of Chemical and Biomolecular Engineering, Georgia Institute of Technology, Atlanta, GA 30332, USA; lingdeer@gmail.com; 2 Department of Biomedical Engineering, University of Massachusetts Lowell, Lowell, MA 01854, USA; ZhenglunAlan_Wei@uml.edu; 3 Wallace H. Coulter Department of Biomedical Engineering, Georgia Institute of Technology & Emory University, Atlanta, GA 30332, USA; 4 Division of Cardiology, Children’s Hospital of Philadelphia, Philadelphia, PA 19104, USA; FOGEL@email.chop.edu; 5 Department of Mathematics and Computer Science, Emory University, Atlanta, GA 30322, USA; ale@mathcs.emory.edu

**Keywords:** congenital heart defects, computational biomechanics, fluid-structure interaction

## Abstract

**Simple Summary:**

A fluid-structure interaction (FSI) simulation of an intra-atrial Fontan connection was performed. Power loss and pressure drop results fluctuated less during the FSI simulation than during the simulation run with rigid walls, but there were no observable differences in time-averaged pressure drop, connection power loss or hepatic flow distribution. These results suggested that employing a rigid wall is a reasonable assumption when evaluating time-averaged hemodynamic quantities of the Fontan connection under resting breath-held flow conditions.

**Abstract:**

Total cavopulmonary connection (TCPC) hemodynamics has been hypothesized to be associated with long-term complications in single ventricle heart defect patients. Rigid wall assumption has been commonly used when evaluating TCPC hemodynamics using computational fluid dynamics (CFD) simulation. Previous study has evaluated impact of wall compliance on extra-cardiac TCPC hemodynamics using fluid-structure interaction (FSI) simulation. However, the impact of ignoring wall compliance on the presumably more compliant intra-atrial TCPC hemodynamics is not fully understood. To narrow this knowledge gap, this study aims to investigate impact of wall compliance on an intra-atrial TCPC hemodynamics. A patient-specific model of an intra-atrial TCPC is simulated with an FSI model. Patient-specific 3D TCPC anatomies were reconstructed from transverse cardiovascular magnetic resonance images. Patient-specific vessel flow rate from phase-contrast magnetic resonance imaging (MRI) at the Fontan pathway and the superior vena cava under resting condition were prescribed at the inlets. From the FSI simulation, the degree of wall deformation was compared with in vivo wall deformation from phase-contrast MRI data as validation of the FSI model. Then, TCPC flow structure, power loss and hepatic flow distribution (HFD) were compared between rigid wall and FSI simulation. There were differences in instantaneous pressure drop, power loss and HFD between rigid wall and FSI simulations, but no difference in the time-averaged quantities. The findings of this study support the use of a rigid wall assumption on evaluation of time-averaged intra-atrial TCPC hemodynamic metric under resting breath-held condition.

## 1. Introduction

The Fontan procedure is a common palliation for patients with single ventricle heart defects [1]. It is usually completed by constructing an intra-atrial tunnel or using an extra-cardiac connection from the inferior vena cava to the pulmonary arteries as the Fontan pathway (FP). Together with the superior anastomosis, this forms the total cavopulmonary connection (TCPC). In all cases, the resulting geometries and constitutive materials can be very different. An intra-atrial TCPC is more bulgy and compliant at the intra-atrial tunnel where vena caval flows mix and re-circulate prior to entering the PAs [2,3]. An extra-cardiac TCPC is composed of a stiffer cylindrical synthetic graft (e.g., Gore-Tex and Dacron grafts), so flow is more streamlined towards the PAs [4]. Even though the TCPC procedure results in favorable short-term outcomes, the patients remain at risk for long term complications [5]. It has been suggested that some of these complications may be attributed to the unfavorable hemodynamics in the connection [6]. For example, there has been evidence showing the possible link between TCPC energy dissipation and patient exercise tolerance [7,8,9,10]. Also, unbalanced distribution of hepatic blood flow between the two sides of the lungs has been associated with the risk of pulmonary arteriovenous malformations [11,12].

Computational fluid dynamics (CFD) serves as a valuable tool to resolve the complex flows in the TCPC, and to understand the hemodynamics of the two types of connections [13,14,15,16,17,18,19,20,21]. CFD analysis allows for a more detailed analysis of flow structures (i.e., vortices, streamlines, pathlines, stagnation points, etc.), flow distributions, pressure distributions, and mechanical stresses (e.g., wall shear stress) than in vitro or in vivo analyses. To simplify analysis and reduce computational cost, previous studies applied various modeling assumptions, e.g., idealized geometries, rigid wall models and steady flow boundary conditions. Recent advancements in image processing technology and computational algorithms have helped addressing some of these assumptions and consider patient’s characteristics in CFD simulation regarding TCPC hemodynamics [22,23,24,25,26,27,28,29]. These studies shifted the computational modeling paradigm to more accurately understand and simulate TCPC hemodynamics.

One limitation of these computational models is the assumption of rigid walls. It has been understood that the expansion and contraction of blood vessels contribute to blood pumping in the body. Bazilevs et al. [30], studied the hemodynamic efficiency differences of realistic extra-cardiac TCPC geometries between rigid and deformable walls using prescribed wall thickness, which demonstrated the difference in resting and exercise hemodynamics between the rigid wall and fluid-structure interaction (FSI) analysis. Orlando et al. [31], carried out a similar analysis using an idealized TCPC model with prescribed material properties and flow rates in the vena cavae, left and right pulmonary arteries, and suture lines, after which they found that the deformable model has 10% higher power loss than the rigid model. Long et al. also performed an FSI CFD analysis of two extra-cardiac TCPCs with varying wall properties for different vessels [21]. The aforementioned studies established the difference between hemodynamics in rigid and deformable TCPC models, but their clinical relevance is still to be investigated, as the prescribed wall properties are yet to be validated. In addition, only idealized TCPCs and extra-cardiac TCPCs were investigated so far. The impact of wall deformation on an intra-atrial TCPC is not well understood.

The objective of this work is to quantify the difference in TCPC hemodynamics between rigid and compliant walls for an intra-atrial TCPC by using the FSI simulation. The wall deformation obtained from the FSI simulations will be compared with the in vivo wall deformation. Finally, the qualitative and quantitative differences of TCPC hemodynamics between rigid and compliant wall conditions will be compared.

## 2. Materials and Methods

### 2.1. Patient Image Acquisition and Reconstruction

**Figure 1:**
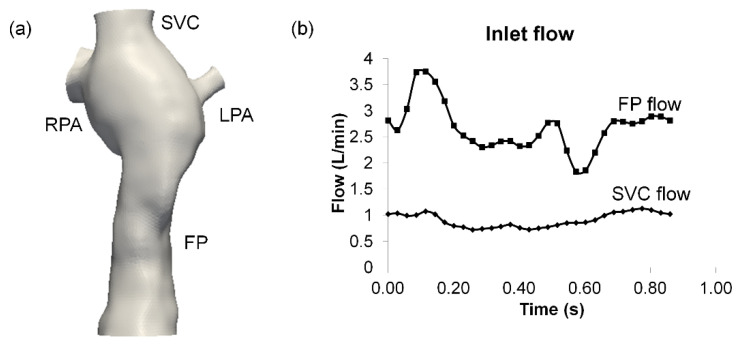
Reconstructed (**a**) 3D anatomy and (**b**) inlet flow waveforms. FP: Fontan pathway, SVC: superior vena cava, LPA/RPA: left/right pulmonary artery.

**Figure 2:**
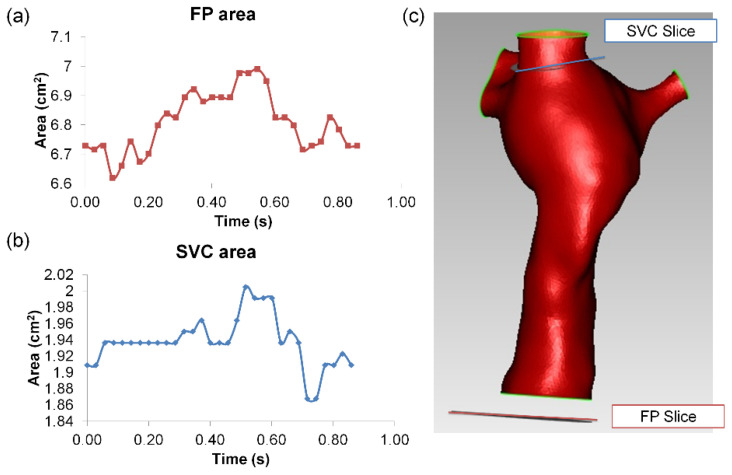
Segmented vessel area waveform of (**a**) FP and (**b**) SVC from phase-contrast MR images. The relative orientation of the slices relative to the TCPC anatomy is shown in (**c**). Note the location of the FP phase-contrast MRI slice was outside the CFD domain. FP: Fontan pathway, SVC: superior vena cava.

**Figure 3:**
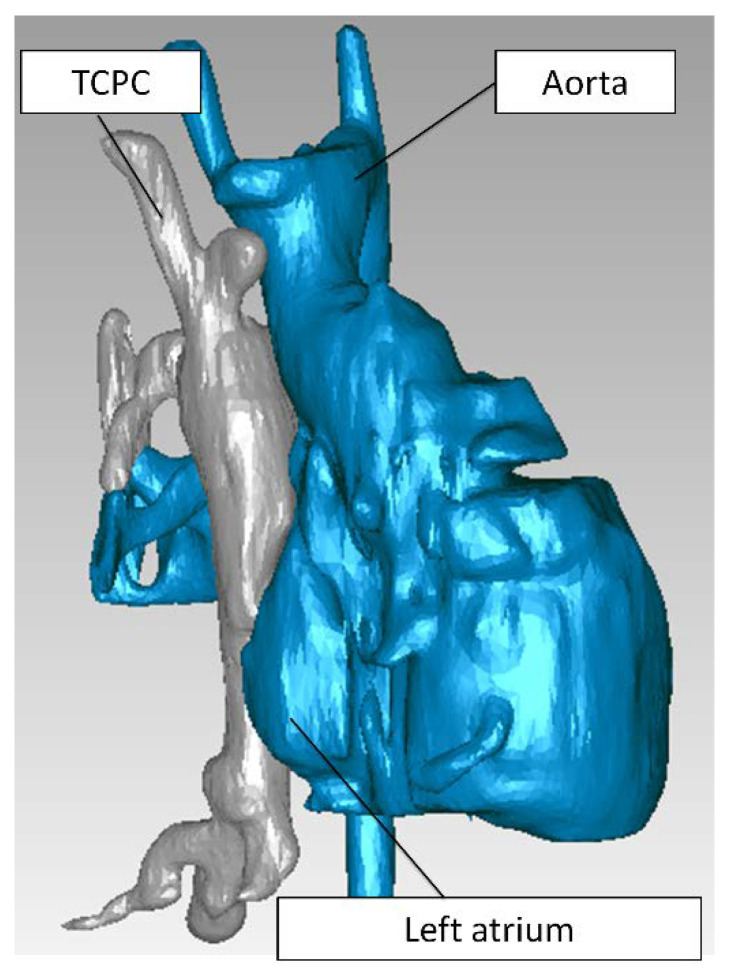
The TCPC (**gray**) and the surrounding heart and blood vessels (**blue**). TCPC: total cavopulmonary connection.

Single ventricle patients with a TCPC anatomy were selected from the Georgia Tech/Children’s Hospital of Philadelphia Fontan database. With informed consent and Institutional Review Board approval, an intra-atrial patient was selected, based on the following criteria: (i) single superior vena cava (SVC) and (ii) no apparent vessel stenosis. Anatomic and phase-contrast magnetic resonance imaging (MRI) acquisition was performed on the patient. Static, steady-state free precession imaging was used to acquire patient-specific anatomic images and 3D anatomies were reconstructed [32,33] (Figure 1a). Phase-contrast MRI was used to acquire through-plane velocity profiles across the vena cavae over a cardiac cycle under resting breath-held conditions. Patient-specific flow conditions were obtained by segmenting phase-contrast MR images at the inlet’s cross-section [34,35]) (Figure 1b). The change in vessel cross-sectional area was also obtained from the segmented phase-contrast MRI slices [36] (Figure 2a,b). The approximate location of the phase-contrast MRI slice relative to the anatomy is shown in (Figure 2c). To visualize the location of the atrial wall relative to the TCPC, the heart, and surrounding pulmonary veins were segmented (Figure 3) with Invesalius 3.0 (http://www.cti.gov.br/invesalius/).

### 2.2. Hemodynamic Assessment

The finite element method solver LifeV (www.lifev.org) was used in this work. The FSI solver is presented in Passerini et al. and has been validated with experimental data of the propagation of a pressure wave in a fluid-filled elastic cylindrical tube [37]. The structural model is based on the assumption of a linear elastic model. Although this is certainly a simplification of the real constitutive law for the vessel wall, it provides a reasonable starting point, which is however, quite indicative for the purposes of this research. The interaction between fluid and structure domains is implemented by the arbitrary Lagrangian-Eulerian approach.

Flow extensions of 2 cm were added to each inlet and outlet for flow development. The resulting surface was loaded into GAMBIT/ANSYS Workbench (ANSYS, Inc., Canonsburg, PA, USA) for surface meshing with unstructured triangular elements. Gmsh [38] was used to prepare volume meshes for both fluid and structure simulations based on the surface mesh. Gmsh preserves the nodes of the input surface mesh when creating a 3D volume mesh. Tetrahedral elements were created in the fluid domains while maintaining the nodes at the input surface. For the structural mesh, the input surface mesh was extruded based on the normal of each element on the surface mesh. It contains two layers of tetrahedral elements. P2 finite elements were used for fluid and structure velocity. P1 finite elements were used for fluid pressure. Notice that with this choice, the meshes for fluid and structures are conformal. This means that the degrees of freedom at the interface between the two domains coincide, and no special matching procedures are required when solving the FSI problem. The duration of one cardiac cycle was 0.86 s, obtained from the MRI data. A timestep of 5 × 10^−4^ s was used at least for three cycles for both rigid wall and FSI simulations.

For the fluid domain, the inflow waveform segmented from phase-contrast MRI was applied as inlet flow boundary conditions. The parabolic velocity profile was assumed at both inlets. The flow extensions have the role of mitigating the impact of the arbitrary choice of a velocity profile at the boundary to fill the mismatch between available data (the flow rate) and velocity conditions required by the mathematical model. In addition, traction-free outflow boundary conditions were used. Blood viscosity and density were assumed to be 3.5 × 10^−6^ m^2^/s and 1000 kg/m^3^, respectively. The same inflow and outflow boundary conditions were applied for FSI and rigid wall simulations. For the structural domain, edges at all inlets and outlets were fixed. The mesh extensions guarantee for this part of the problem that the arbitrary displacement conditions have a limited impact on the solution in the region of interest. The external side of the wall was allowed to move freely throughout the simulations. Poisson’s ratio of 0.3 and a wall thickness of 2.0 mm was prescribed. An iterative method [39] was employed to estimate Young’s modulus of TCPC, resulting in consistent deformation indices between the FSI simulation and in vivo measurement. The deformation index was computed to quantify the amplitude of cross-sectional area change at the FP and the SVC since they are the more compliant vessels of the TCPC:

$$Deformation\_Index=\frac{A_{max}-A_{min}}{A_{mean}}\times 100\%$$

The estimated Young modulus was 0.07 MPa, which fell into ranges of literature values for blood vessel walls [40,41,42,43,44]. Homogeneous material properties were assigned at the vessel wall. This study’s primary hemodynamic metrics are power loss and hepatic flow distributions: their definitions adhere to previous studies [23,45,46,47,48].

## 3. Results

### 3.1. Mesh Sensitivity

To investigate mesh sensitivity, three meshes were created and simulated with the same boundary conditions:

(a) Very fine mesh—1 mm mesh edge length
  - Fluid: 193,974 elements
  - Structure:164,178 elements
(b) Fine mesh—1.5 mm mesh edge length
  - Fluid: 97,793 elements
  - Structure: 82,206 elements
(c) Coarse mesh—2 mm mesh edge length
  - Fluid: 27,640 elements
  - Structure: 44,406 elements

**Figure 4:**
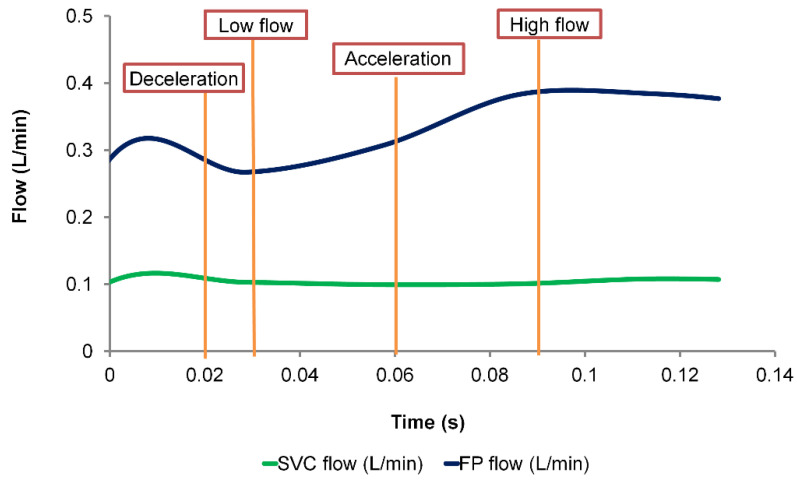
Low waveform of the mesh sensitivity study. Maximum mesh displacement was extracted at four phases (deceleration, low flow, acceleration and high flow) and compared between the different mesh sizes. FP: Fontan pathway, SVC: superior vena cava.

Because the very fine mesh requires much higher computational time, a time duration of 0.13 s was simulated with all three meshes and compared. Mesh displacements at four phases (deceleration, low flow, acceleration, and high flow) were extracted (Figure 4).

Comparing the maximum displacement between the different mesh sizes, they are all of the similar magnitudes (Table 1). The discrepancy of maximum displacement between very fine mesh and fine mesh ranged from 0.002 mm to 0.031 mm. Comparing the fine mesh and the coarse mesh, the discrepancy ranged from 0.0 mm to 0.045 mm.

Comparing the pressure drop differences at each time step, the average (temporal) difference between the coarse and the fine mesh was 0.009 mmHg, and the maximum difference was 0.015 mmHg. The average (temporal) pressure drop difference between fine and very fine mesh was 0.005 mmHg. The maximum pressure drop difference was 0.01 mmHg. Comparing time-averaged power loss over the simulated time span, there was a 0.66% difference between the coarse mesh and the fine mesh, and a 0.29% difference between the fine mesh and the very fine mesh (Table 2). Simulations with the very fine mesh are computationally expensive. The hemodynamic and wall displacement predictions with the fine mesh are in close proximity to the very fine mesh prediction. Therefore, in the following sections, the fine mesh is used throughout this study. Moreover, in the following sections, the cardiac cycle starts at t=0.

### 3.2. Simulated TCPC Wall Deformation

**Figure 5:**
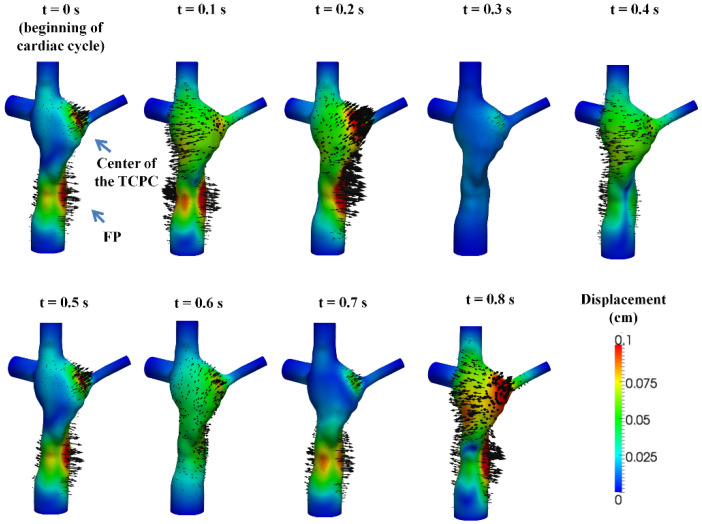
The simulated displacement fields at nine evenly spaced time points in the cardiac cycle. The color of the contour represents the magnitudes of the displacement and the arrows represent the direction of the displacement.

The simulated TCPC wall displacement is shown in Figure 5. Wall displacement magnitude increases from t = 0 s to t = 0.2 s, expanding the TCPC volume. The maximum wall displacement of 0.21 cm occurred at t = 0.165 s, which was 0.065 s after the time point of maximum FP flow (t = 0.1 s). At t = 0.3 s, wall displacement was almost zero. At that time point, the TCPC volume (62.49 mL) was almost equal to the volume of the rigid wall simulation fluid domain (62.25 mL). After that time point, the wall displacement magnitudes of both the FP and the center of the TCPC increased from t = 0.4 s to 0.5 s. Wall displacement magnitude then decreased at t = 0.6 s. At t = 0.7 s, wall displacement mainly occurred at the FP. At t = 0.8 s, wall displacement occurred at both the FP and the center of the TCPC, expanding the TCPC volume. The simulated wall displacement animations are available in the Supplementary Materials section of the journal online.

**Figure 6:**
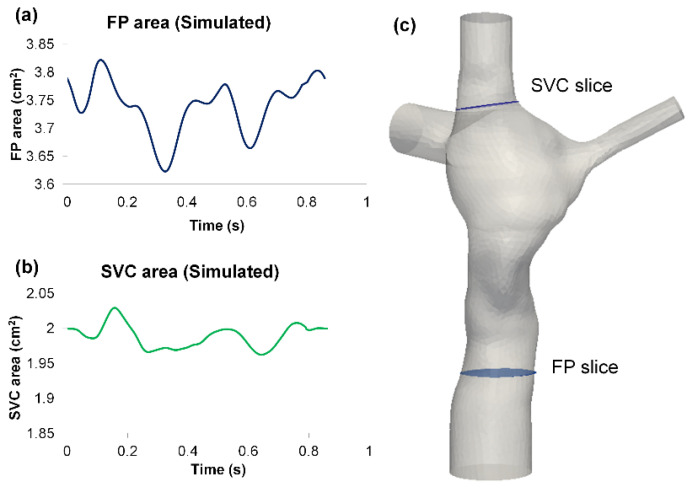
Simulated vessel area waveform of: (**a**) FP and (**b**) SVC from the FSI simulation results. The “FP slice” and “SVC slice” in (**c**) represent the location of where the FP and SVC area waveforms were extracted. FP: Fontan pathway, SVC: superior vena cava.

From the FSI simulation results, the cross-sectional vessel areas of FP and SVC were extracted throughout the simulated cardiac cycle (Figure 6). The vessel areas were compared between FSI and phase-contrast MRI data (Table 3). Since the location of the phase-contrast MRI slice of the FP was outside the CFD domain, a comparison of absolute values of wall displacement is not feasible; therefore, the deformation index was used for comparisons instead. Simulation results showed that deformation index from the simulation was in close agreement with the deformation index of the phase-contrast MRI data at the FP. For the SVC, the maximum and average areas were similar between FSI and phase-contrast MRI, but the deformation index was underestimated in the simulation since the simulated minimum SVC area was higher.

**Figure 7:**
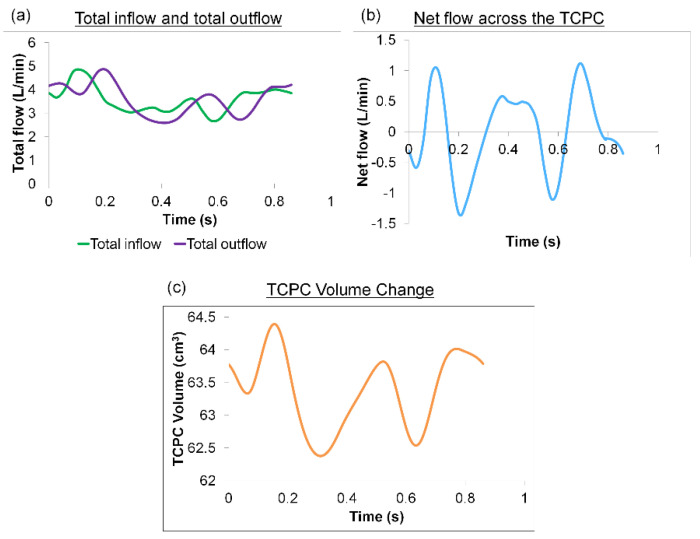
(**a**) Waveforms of the total inflow and outflow (**b**) Waveform of the net flow through the TCPC (**c**) Waveform of the TCPC volume throughout the cardiac cycle. TCPC: total cavopulmonary connection.

The net flow (total inflow−total outflow) through the TCPC was computed from the FSI simulations. The TCPC volume change was then computed by integrating the net flow over time using the trapezoidal rule (Figure 7). Throughout the simulated cardiac cycle, the minimum TCPC volume was 62.49 cm^3^, and the maximum volume was 64.46 cm^3^. This means the TCPC changed its volume by 1.97 mL throughout the cycle. From the FSI simulation results, the maximum and minimum pressure (averaged over the entire TCPC volume) was 0.87 mmHg and 0.07 mmHg, respectively. The change in FP pressure was, therefore, 0.8 mmHg throughout the cycle. By dividing the maximum volume change (1.97 mL) by maximum TCPC pressure change (0.8 mmHg), TCPC compliance was estimated to be 2.46 mL/mmHg.

### 3.3. TCPC Flow Field

**Figure 8:**
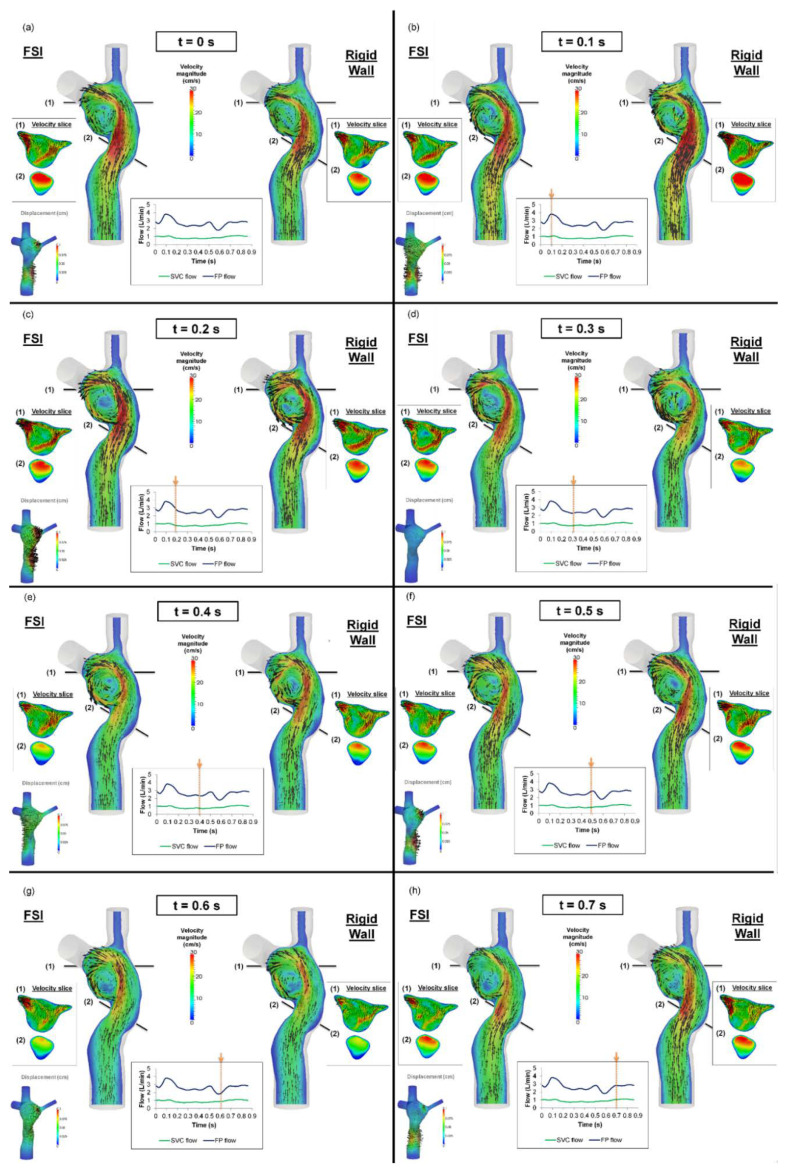
Flow field of the TCPC from the fluid-structure interaction (FSI) and rigid wall simulations (**a**–**h**) from t = 0 s to t = 0.7 s, at a 0.1 s interval. The displacement field of the FSI simulation at the corresponding time point is shown in the bottom-left corner. FP: Fontan pathway, SVC: superior vena cava, TCPC: total cavopulmonary connection.

The flow fields throughout eight evenly spaced time points of the cardiac cycle are shown in Figure 8. Two planes were extracted at (1) the center of the TCPC and (2) across the FP. It is interesting to note the high degree of similarity between the FSI and rigid wall simulation results. The main differences between the two flow fields are at the center of the TCPC and the FP flow jet, in which the velocity jets are shown in the two extracted planes. At t = 0 s, the volume of the TCPC expanded in the FSI simulation. The FP jet has a higher maximum velocity in the FSI simulation than the rigid wall simulation. At t = 0.1 s, the FP continues to expand. However, at this time point, the FP jet carried a higher maximum velocity in the rigid wall than the FSI simulation. At t = 0.2 s, the wall deformation occurred at both the FP and the center of the TCPC. The FP jet trajectory and velocity magnitude were similar between the two simulations. However, at the center of the TCPC, the velocity magnitudes and directions were different between the FSI and rigid wall simulations. At t = 0.3 s, the FSI and rigid wall simulations have similar total TCPC volumes. However, the maximum velocity magnitude of the FP jet was lower under rigid wall conditions. Also, the velocity magnitudes were different at the center of the TCPC. At t = 0.4 s, the TCPC continued to expand again in the FSI simulation. The maximum velocity was lower in the FSI simulation at this time point. At t = 0.5 s, both the FP and the center of the TCPC expanded, affecting the velocity magnitude in the FSI simulation. This also affected the velocity magnitude and direction at the center of the TCPC. From t = 0.5 s to the end of the cycle, as the wall displacement fluctuated, the velocity magnitude and direction were different between the FSI and rigid wall simulations at the center of the TCPC.

### 3.4. Pressure Drop and TCPC Power Loss

**Figure 9:**
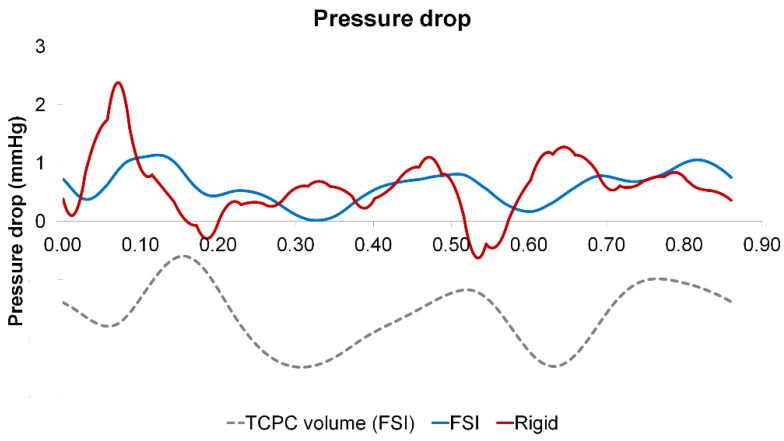
Pressure drop waveforms of the rigid wall and fluid-structure interaction (FSI) simulations within a cardiac cycle. The waveform of the TCPC volume from the FSI simulation is shown simultaneously. TCPC: total cavopulmonary connection.

**Figure 10:**
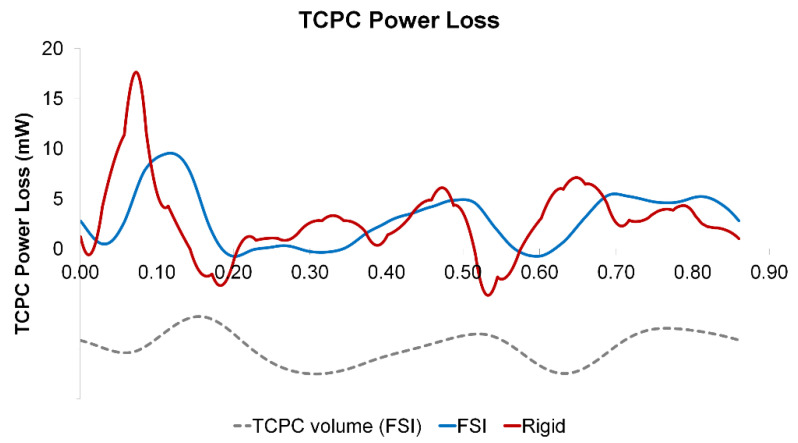
TCPC power loss waveforms of the rigid wall and fluid-structure interaction (FSI) simulations within a cardiac cycle. The waveform of the TCPC volume from the FSI simulation is shown simultaneously. TCPC: total cavopulmonary connection.

The instantaneous pressure drops (FP to left pulmonary artery pressure) and power loss across the TCPC in the cardiac cycle are shown in Figure 9 and Figure 10, respectively. The pressure drop and power loss waveforms of the rigid wall and FSI simulations shared similar shapes, while the waveforms of the FSI simulation lag behind that of the rigid wall simulation. Comparing the maximum and minimum pressure drops and power losses, the rigid wall simulation has larger fluctuations than that of the FSI simulation. The maximum pressure drop and maximum power loss were lower in the FSI simulation, which is likely to be due to the increase of the TCPC volume in the FSI simulation. When comparing the time-averaged pressure drop and power loss, the differences between the two simulations were small (pressure drop difference = 0.01 mmHg, TCPC power loss difference = 0.1 mW) (see Table 4).

### 3.5. Particle Tracking

To assess the impact of wall deformation on particle residence times and %HFD(LPA) (the hepatic flow distribution to the left pulmonary artery), a Lagrangian particle tracking analysis was performed with ParaView software (Kitware Inc., Clifton Park, NY, USA). For each condition, approximately 700 (number of nodes at the FP cross-sections) particles were seeded at the FP at every 0.001 s for one cardiac cycle (0.86 s) and were passively advected with the flow for five additional cardiac cycles.

**Figure 11:**
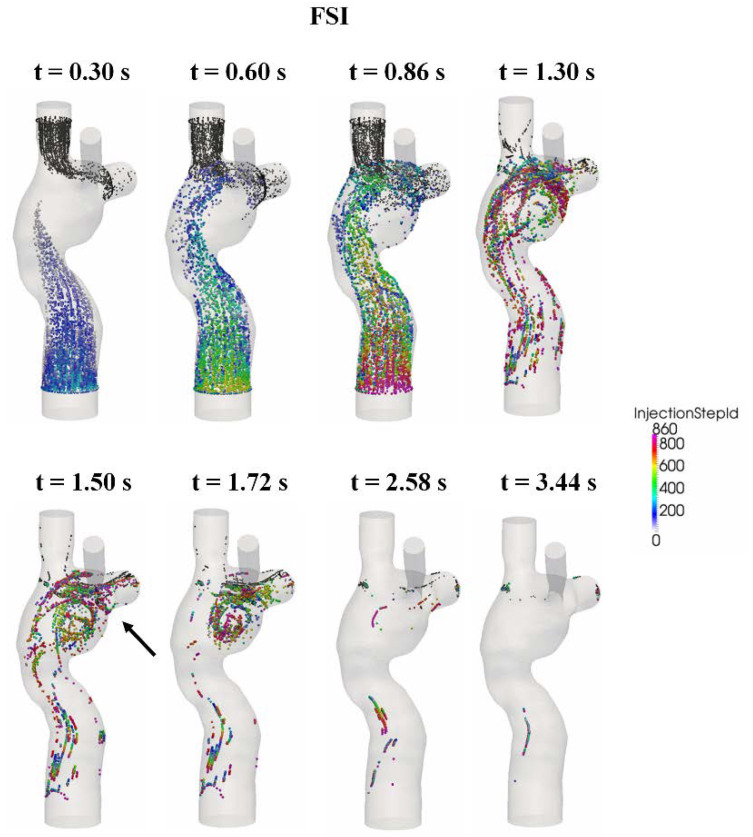
Progression of the particles seeded at the FP from the fluid-structure interaction (FSI) simulation. The FP particles are color coded by their seeding time step. SVC particles are colored black. FP: Fontan pathway, SVC: superior vena cava.

**Figure 12:**
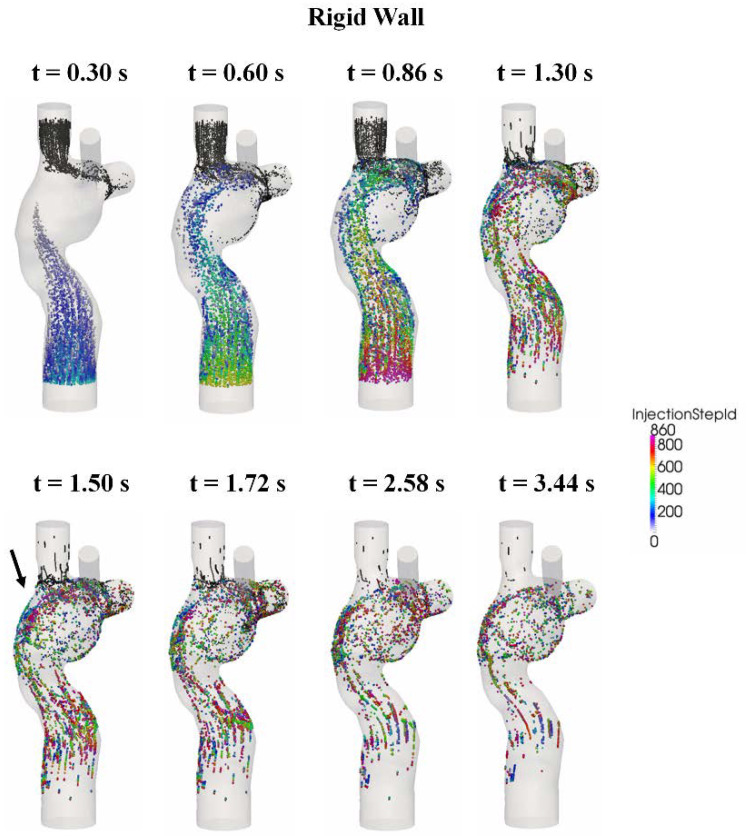
Progression of the particles seeded at the FP from the rigid wall simulation. The FP particles are color-coded by their seeding time step. SVC particles are colored in black. FP: Fontan pathway, SVC: superior vena cava.

The particle trajectories within the flow fields are shown in Figure 11 (FSI) and Figure 12 (rigid wall) for eight time points, and the corresponding animations are available in the supplementary material section of the journal online. The FP particles are colored based on the time at which they were seeded, while the SVC particles are colored in black. There is no major difference in the particle trajectory at the beginning of the filling phase (t = 0.3 s) between the two wall conditions. Under conditions, SVC and FP flow met at the neck of the SVC-PA junction (t = 0.6 s), and together they circulated at the middle of the TCPC (t = 0.86 s).

After the second cycle (t > 0.86 s), the particles circulate before leaving the domain under both FSI and rigid wall conditions (t = 1.3 s). However, the particle washout trajectories were different in the FSI and rigid wall simulations. From t = 1.5 s to t = 1.72 s, the particles circulated at the center of the TCPC close to the PAs for the FSI simulation. For the rigid wall simulation from t = 1.5 s to t = 1.72 s, the particles circulated within the entire region of the center of the TCPC, instead of just close to the PAs. From t = 2.58 s to t = 3.44 s, most particles had already exited the domain in the FSI simulation. For rigid wall simulation from t = 2.58 s to t = 3.44 s, particles still remained in the domain. Many of these particles are found close to the walls of the TCPC.

**Figure 13:**
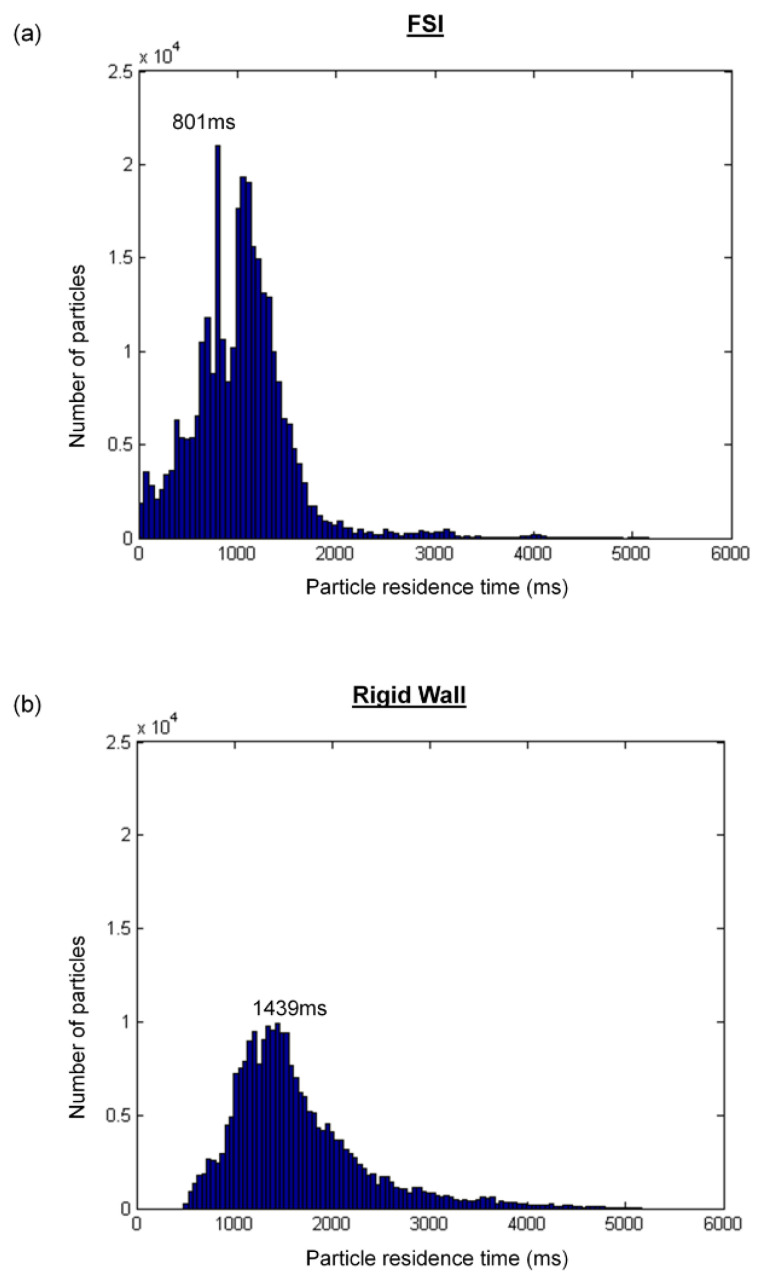
Distribution of the residence times for particles injected at the inferior vena cava: (**a**) fluid-structure interaction (FSI) simulation and (**b**) rigid wall simulation.

From Figure 11, it can be seen that very few particles remain in the domain after one cardiac cycle (FSI simulation). It is found that with a deformable wall, most of the particles from the FP left the domain within one heartbeat. The quantitative distribution of the FP particle residence times is shown in Figure 13 and is compared between the FSI and rigid wall simulations. The particle residence time distributions are very different. The peak of the distribution of the FSI simulation occurred at 801 ms, whereas that for the rigid wall simulations occurred at 1439 ms. Also, the peak of the rigid wall simulation is of smaller amplitude. A majority of particles seeded at the FP in the rigid wall simulation took more than one cardiac cycle (0.86 s) to leave the TCPC. The particle washout time (time at which 95% of the FP particles left the domain) is shown in Table 5. It took 1.77 s (~2 cardiac cycle) for 95% of the FP particles to leave the TCPC for the FSI simulation. It took much longer (3.16 s) for 95% of the FP particles to exit the domain for the rigid wall simulation.

**Figure 14:**
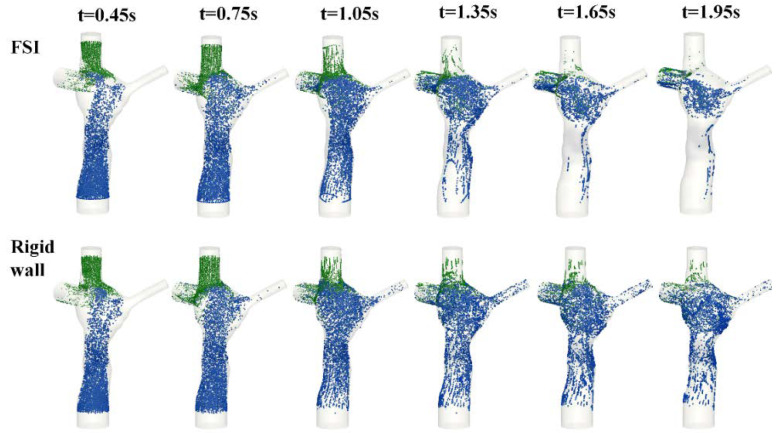
Screenshots of the particle tracking results of fluid-structure interaction (FSI) and rigid wall simulations from t = 0.45 s to t = 1.95 s. Particles originating from the FP are colored blue, and particles originating from the SVC are colored green. FP: Fontan pathway.

**Figure 15:**
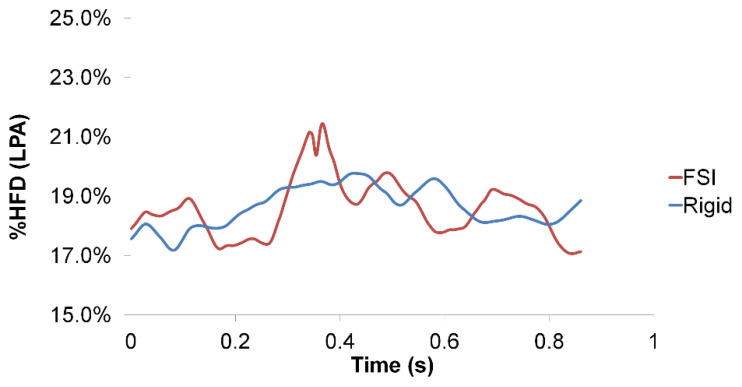
Hepatic flow distribution to the left pulmonary artery, %HFD(LPA), of the rigid wall and fluid-structure interaction (FSI) simulations within a cardiac cycle.

Particle tracking was also performed by visualizing the particles based on the vessel of origin. Figure 14 illustrates that, in both the FSI and rigid wall simulations, the majority of the SVC particles exited through the right pulmonary artery due to proximity. Even with the differences in the velocity magnitude of the FP jet and velocity field at the center of the TCPC as observed earlier, there was little difference in the FP particle trajectory between FSI and rigid wall simulations. From the particle tracking results, HFD was also computed. Instantaneous %HFD(LPA) is shown in Figure 15. It is observed that %HFD to the left pulmonary artery fluctuated in time more in the FSI simulation than the rigid wall simulation. However, comparing the time-averaged %HFD(LPA), no difference is found between the two conditions, Table 5.

## 4. Discussion

In most CFD models of the TCPC, a rigid wall is often assumed for simplicity and reduced computational cost. However, in reality, the TCPC is composed of native tissues (caval veins and pulmonary arteries), which are compliant. Knowing that TCPC vessel wall deforms in vivo, it is important to understand how such deformation can affect hemodynamics. Long et al. performed FSI CFD analysis on two extra-cardiac TCPCs with varying wall properties [21]. The results showed that there was little effect of FSI (with both homogenous and heterogeneous vessel wall material properties) on pressure tracings, HFD, and time-averaged energy efficiency. However, the effect of FSI on instantaneous energy efficiency and wall deformation was significant. This study highlighted that the impact of FSI on TCPC hemodynamics is relevant to the metric of interest. However, several assumptions were made with regards to the wall material properties, which have not been validated. Also, this study was performed on extra-cardiac patients only. The geometry and constitutive materials of the extra-cardiac and intra-atrial TCPC can be very different. It is expected that the difference in flow dynamics between the rigid wall and compliant wall simulations to be more profound in the intra-atrial TCPCs. Mirabella et al. investigated the effect of wall deformability on intra-atrial TCPC hemodynamics with in vivo wall deformation data [49]. Using cine anatomic MRI data, the in vivo wall deformation was prescribed in a CFD model that includes a moving domain. The largest differences between rigid and moving wall models were observed in measures of energetic efficiency of TCPC as well as in hepatic flow distribution and transit time of seeded particles through the connection. This study highlighted the importance of wall deformation on intra-atrial TCPC hemodynamics. However, this approach is not necessarily applicable for prospective modeling, as one cannot always predict how the vessel wall will deform after the surgical connection is being altered.

In the current study, the simulated wall deformation of the FP and SVC was compared with phase-contrast MRI data. Using a normalized metric, the deformation index, the change in vessel area at the FP and SVC were compared between the numerical simulation and phase-contrast MRI data to validate the FSI simulations. It was found that the FP deformation index was in a close agreement between FSI simulation and in vivo data. This is important as intra-atrial FP wall deformation was the focus of this work. This suggested the assigned wall material property at the FP was a reasonable estimation of the in vivo wall properties. However, the SVC deformation index agreement between FSI simulation and phase-contrast MRI was not optimal. This could be attributed to the following reasons: It was possible that the assigned homogenous material properties throughout the TCPC were different from the in vivo SVC wall material property. In reality, the material properties in FP and SVC can be different. From the change in TCPC volume as well as the change in pressure, TCPC compliance was estimated to be 2.46 mL/mmHg. This is in the same order of magnitude as PA compliance in healthy subject = 2.87 mL/mmHg [50].

Qualitative differences in the TCPC flow field were observed at the FP velocity jet and the center of the TCPC between the rigid wall and FSI simulations. These were also the regions in which most wall displacements were observed in the FSI simulation. Particle residence time provides a quantitative measure for the differences of particle pathways under the rigid wall and compliant wall conditions. The longer residence times of the rigid wall condition were mostly associated with FP particles evolving at a low velocity close to the rigid wall (Figure 12). On the other hand, with a deformable wall, the particles in the wall boundary layers had a higher probability of being redirected towards the bulk of the flow during contraction of the deforming wall.

TCPC pressure drop and power loss are important hemodynamic metrics, as high power loss suggests a less efficient pathway, which has been related to patient exercise intolerance [8,9,10,51]. Pressure drop and power loss were compared between the rigid wall and FSI simulations. Instantaneous pressure drop and power loss vary between FSI and rigid wall simulations. Comparing the maximum and minimum pressure drops and power loss, the rigid wall simulation has larger fluctuations than that of the FSI simulation, which is in agreement with a previous similar study on the carotid artery [52]. However, the differences in time-averaged pressure drops (0.01 mmHg) and power losses (0.1 mW) between the two simulations were small. Hepatic flow distribution was also quantified, which is a TCPC hemodynamic metric that was related to the risk of pulmonary arteriovenous malformations [11,12,53]. As shown in Figure 15, the instantaneous HFD varies within the simulated cycle between the rigid wall and FSI simulations. However, there was no difference in time-averaged HFD between the two conditions, which is in agreement with Long et al. [21].

Previously, it was speculated that the hemodynamic differences observed in intra-atrial and extra-cardiac TCPCs were attributed to its geometry as well as wall properties. From this work, it was found that wall compliance has little influence on time-averaged hemodynamic quantities under resting breath-held conditions. This also means that the wall compliance of the intra-atrial TCPC did not contribute significantly to TCPC hemodynamics as one would be expected.

The results in this work suggest that wall deformation has an impact on the instantaneous hemodynamic metrics of the TCPC (instantaneous flow field, particle trajectory, pressure drop, power loss and HFD). However, FSI has little impact on time-averaged quantities (pressure drop, power loss, HFD) under resting breath-held condition. Considering time-averaged hemodynamic quantities are the more common surrogates of patients’ hemodynamic performance for surgical planning of TCPC, the results here support the notion that a rigid wall assumption is a reasonable assumption for such image-based surgical planning systems [29,53,54,55,56]. The framework for a surgical planning system of TCPC already exists [57,58] to prospectively model different surgical configurations. Time-averaged quantifies (mainly power loss and HFD) were compared across different surgical options, usually with resting breath-held flow boundary conditions. A rigid wall assumption was often applied for simplicity in previous computational or experimental research for Fontan patients. It was originally expected that including vessel deformability into the system can possibly help more realistically predicting post-operative scenarios. The results of this work suggest that a rigid wall assumption is a reasonable approximation when evaluating time-averaged quantities under resting breath-held conditions. Especially when the surgery planning needs to be completed in a short time frame, and FSI simulation has a higher computational cost, it could be expected that the error associated with a rigid wall assumption will be minimal.

### Limitations

A linear elastic structural model was assumed for the vessel wall. Stress-free outflow boundary conditions were also used. Also, TCPC is composed of heterogeneous materials. It contains native tissue, as wells as stiffer surgical materials and suture lines. In addition, the initial condition of the FSI simulation used geometry from patient-specific MR images, which represents a deformed configuration stressed by the in vivo conditions. In the simulation, the structure was assumed to be stress-free initially. However, these limitations should not affect the conclusions of this study. In addition, only one patient was included in this study. Future work should extend this study to more patients.

## 5. Conclusions

In this study, the rigid wall assumption used in most CFD models of the TCPC was evaluated. A detailed comparison of TCPC hemodynamics under a rigid wall and a compliant wall condition was performed with respect to the surrogates for TCPC efficiency, namely power loss, HFD, and particle washout time on an intra-atrial patient. The wall deformation in the simulated FP was in the same magnitude as that observed in vivo. The simulation results suggest that a rigid wall assumption appears to provide an adequate representation of the time-averaged TCPC power loss and pressure drop. There was no difference in time-averaged HFD between the rigid wall and FSI simulations. In summary, the results here support the use of a rigid wall assumption on the evaluation of time-averaged TCPC hemodynamic metric under resting breath-held condition.

## Figures and Tables

**Table 1:** Maximum mesh displacement with different mesh sizes.

Maximum Displacement (mm)	(a) Very Fine	(b) Fine	(c) Coarse	Difference Between	
				(a) & (b)	(b) & (c)
Deceleration (at 0.02 s)	2.448	2.450	2.457	0.002	0.007
Low flow (at 0.03 s)	4.334	4.342	4.342	0.008	0
Acceleration (at 0.06 s)	6.955	6.964	6.979	0.009	0.015
High flow (at 0.09 s)	6.886	6.917	6.962	0.031	0.045

**Table 2:** Differences in pressure drop and power loss with different mesh sizes.

Hemodynamic Metrics		(a) Very Fine vs. (b) Fine	(b) Fine vs. (c) Coarse
Pressure drop difference (mmHg)	Temporal average	0.005	0.009
	Temporal maximum	0.01	0.015
%difference in power loss		0.29%	0.66%

**Table 3:** Comparison of vessel areas between fluid-structure interaction (FSI) simulation and in vivo phase-contrast MRI (PC-MRI) data. FP: Fontan pathway, SVC: superior vena cava.

Vessel Area	FSI Simulation		PC-MRI Data	
	FP	SVC	FP	SVC
Average (cm^2^)	3.74	1.99	6.81	1.94
Change (cm^2^)	0.20	0.07	0.37	0.14
Deformation Index	5.3%	3.4%	5.4%	7.1%

**Table 4:** Comparison of pressure drop and TCPC power loss between the rigid wall and fluid-structure interaction (FSI) simulations over the cardiac cycle. TCPC: total cavopulmonary connection.

	Pressure Drop (mmHg)			TCPC Power Loss (mW)		
	Minimum	Maximum	Average	Minimum	Maximum	Average
Rigid wall	−0.60	2.35	0.60	−4.46	17.33	2.89
FSI	−0.07	1.13	0.61	−0.82	9.34	2.99

**Table 5:** Comparison of particle washout time and time-averaged hepatic flow distribution to the left pulmonary artery, %HFD(LPA), between rigid wall and fluid-structure interaction (FSI) simulations.

	Particle Washout Time (s)	Particle Washout Time (No. of Cardiac Cycle)	Time-Average HFD(LPA)
FSI	1.77	2.06	19%
Rigid wall	3.16	3.67	19%

## Supplementary Materials

The following are available online at https://www.mdpi.com/2079-7737/9/12/412/s1. Video S1: Animation of the simulated mesh displacement viewing from the anterior angle. The flow waveform is shown simultaneously, Video S2: Animation of the simulated mesh displacement viewing from the left. The size and color of the arrows represent the magnitude of the displacement, Video S3: Particle tracking video of FSI simulation. Particles are colored by its release time step, Video S4: Particle tracking video of rigid wall simulation. Particles are colored by its release time step

## Nomenclature

TCPC: total cavopulmonary connection; FP: Fontan pathway; CFD: computational fluid dynamics; FSI: fluid-structure interaction; MRI: magnetic resonance imaging; SVC: superior vena cava; HFD: hepatic flow distribution.
